# Structural correlates of language processing in primary progressive aphasia

**DOI:** 10.1093/braincomms/fcad076

**Published:** 2023-03-16

**Authors:** Curtiss A Chapman, Maryna Polyakova, Karsten Mueller, Christopher Weise, Klaus Fassbender, Klaus Fliessbach, Johannes Kornhuber, Martin Lauer, Sarah Anderl-Straub, Albert Ludolph, Johannes Prudlo, Anja Staiger, Matthis Synofzik, Jens Wiltfang, Lina Riedl, Janine Diehl-Schmid, Markus Otto, Adrian Danek, FTLD Consortium Germany, Annerose Engel, Gerdi Pfüller, Daniéle Pino, Frank Regenbrecht, Angelika Thöne-Otto, Timo Oberstein, Bernhard Landwehrmeyer, Jolina Lombardi, Elisa Semler, Jan Kassubek, Gesa Hartwigsen, Matthias L Schroeter

**Affiliations:** Lise Meitner Group for Cognition and Plasticity, Max Planck Institute for Human Cognitive and Brain Sciences, Leipzig 04103, Germany; Department of Neurology, Max Planck Institute for Human Cognitive and Brain Sciences, Leipzig 04103, Germany; Department of Neurology, University of Leipzig Medical Center, Leipzig 04103, Germany; Department of Neurology, Max Planck Institute for Human Cognitive and Brain Sciences, Leipzig 04103, Germany; Department of Neurology, University of Leipzig Medical Center, Leipzig 04103, Germany; Department of Neurology, University of Halle Medical Center, Halle 06120, Germany; Department of Neurology, Saarland University Hospital, Homburg 66421, Germany; Department of Psychiatry and Psychotherapy, University Hospital Bonn, Bonn 53127, Germany; German Center for Neurodegenerative Diseases (DZNE), Germany; Department of Psychiatry and Psychotherapy, University Hospital Erlangen, Erlangen 91054, Germany; Department of Psychiatry and Psychotherapy, University Hospital Würzburg, Würzburg 97080, Germany; Department of Neurology, University of Ulm, Ulm 89081, Germany; Department of Neurology, University of Ulm, Ulm 89081, Germany; German Center for Neurodegenerative Diseases (DZNE), Germany; Department of Neurology, University Medicine Rostock, Rostock 18051, Germany; Clinical Neuropsychology Research Group, Institute of Phonetics and Speech Processing, Ludwig-Maximilians-University Munich, Munich 80539, Germany; German Center for Neurodegenerative Diseases (DZNE), Germany; Department of Neurodegenerative Diseases, Center of Neurology, Hertie Institute for Clinical Brain Research, Tübingen 72076, Germany; German Center for Neurodegenerative Diseases (DZNE), Germany; Department of Psychiatry and Psychotherapy, Medical University Göttingen, Göttingen 37075, Germany; Department of Psychiatry and Psychotherapy, Technical University of Munich, Munich 80333, Germany; Department of Psychiatry and Psychotherapy, Technical University of Munich, Munich 80333, Germany; Department of Neurology, University of Ulm, Ulm 89081, Germany; Department of Neurology, Ludwig-Maximilians-University Munich, Munich 80539, Germany; Lise Meitner Group for Cognition and Plasticity, Max Planck Institute for Human Cognitive and Brain Sciences, Leipzig 04103, Germany; Department of Neurology, Max Planck Institute for Human Cognitive and Brain Sciences, Leipzig 04103, Germany; Department of Neurology, University of Leipzig Medical Center, Leipzig 04103, Germany

**Keywords:** primary progressive aphasia, language, semantics, cortical thickness

## Abstract

Understanding the relationships between brain structure and language behaviour in primary progressive aphasia provides crucial information about these diseases’ pathomechanisms. However, previous investigations have been limited from providing a statistically reliable view of broad language abilities by sample size, variant focus and task focus. In this study, the authors aimed to determine the relationship between brain structure and language behaviour in primary progressive aphasia, to determine the degree to which task-associated regions were atrophied across disease variants and to determine the degree to which task-related atrophy overlaps across disease variants. Participants were 118 primary progressive aphasia patients and 61 healthy, age-matched controls tested from 2011 to 2018 in the German Consortium for Frontotemporal Lobar Degeneration cohort. Diagnosis of primary progressive aphasia required progressive deterioration of mainly speech and language for **≥** 2 years, and variant was diagnosed by the criteria of Gorno-Tempini *et al*. (Classification of primary progressive aphasia and its variants. *Neurology*. 2011;76(11):1006-1014). Twenty-one participants not fulfilling a specific subtype were classified as mixed-variant and excluded. Language tasks of interest included the Boston naming test, a German adaptation of the Repeat and Point task, phonemic and category fluency tasks and the reading/writing subtest of the Aachen Aphasia Test. Brain structure was measured by cortical thickness. We observed networks of language task-associated temporal, frontal and parietal cortex. Overlapping task-associated atrophy was observed in the left lateral, ventral and medial temporal lobes, middle and superior frontal gyri, supramarginal gyrus and insula. Some regions, primarily in the perisylvian region, were associated with language behaviour despite showing no significant atrophy. The results crucially extend less powerful studies associating brain and language measures in primary progressive aphasia. Cross-variant atrophy in task-associated regions suggests partially shared underlying deficits, whereas unique atrophy reinforces variant-specific deficits. Language task-related regions that are not obviously atrophied suggest regions of future network disruption and encourage understanding of task deficits beyond clearly atrophied cortex. These results may pave the way for new treatment approaches.

## Teaser

In 118 patients with primary progressive aphasia, Chapman *et al*. found that language tasks associated with cortical thickness in temporal, frontal and parietal brain regions. Cross-variant atrophy suggested shared underlying deficits; unique atrophy reinforced variant-specific deficits; and unatrophied, language-related regions suggested regions of future network disruption.

## Graphical Abstract

**Graphical Abstract:**
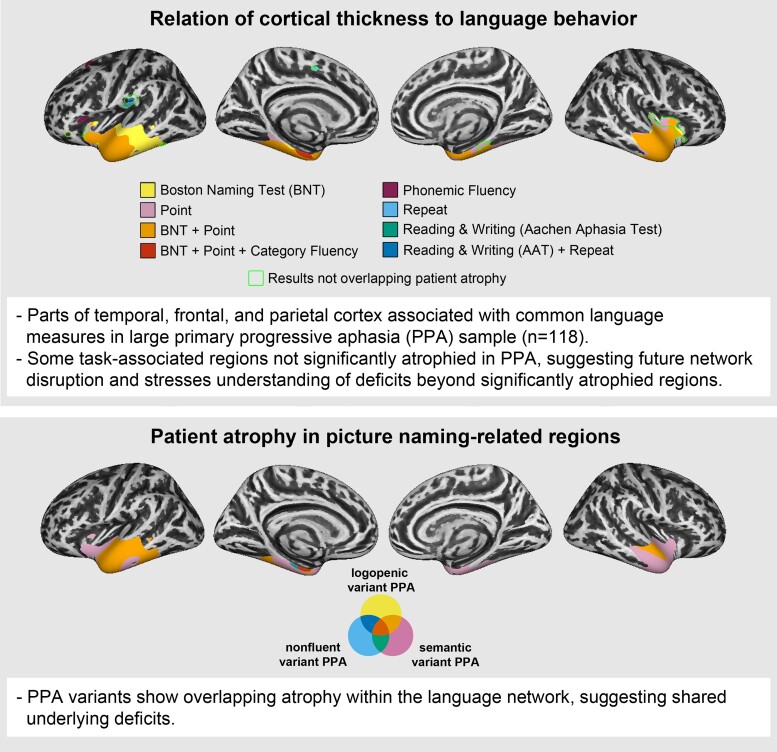

## Introduction

Primary progressive aphasia (PPA) is a neurodegenerative clinical syndrome in which brain atrophy is associated with progressive language deficits with relative sparing of other cognitive abilities in early stages of the disease.^1^ Three variants of PPA are recognized in the literature, each of which shows a distinguishable core set of language deficits and pattern of cortical degeneration.^2,3^ The logopenic variant (lvPPA) presents with impaired sentence and phrase repetition and single-word retrieval issues along with peak atrophy to left posterior perisylvian temporal and parietal cortex. The nonfluent-agrammatic variant (nfvPPA) is characterized by agrammatic language production or effortful speech along with peak atrophy to left frontal and insular regions. The semantic variant (svPPA) shows impaired single word comprehension and anomia along with peak atrophy to the left anterior temporal lobe (ATL). As the disease progresses, the variants become more similar in behaviour and cortical etiology.^4^

The relationship between cortical atrophy and speech, language and cognitive measurements in PPA is both clinically and theoretically important, as it helps to delineate pathomechanisms of the different PPA subtypes and informs how the healthy language network works. For example, previous research shows that sentence repetition in lvPPA is related to left superior temporal lobe,^5,6^ agrammatism in nfvPPA is related to left inferior frontal lobe,^5,7^ and semantic processing in svPPA is related to bilateral ATLs.^8^ Unfortunately, the few existing studies have had limited power to detect anatomical–behavioural relationships due to small sample sizes. Consequently, common and divergent anatomical underpinnings of behavioural deficits on the same tasks remain to be verified and uncovered, which would allow for comprehensive protocols treating unique and shared deficits across PPA variants. Previous studies also often narrowly focus on one PPA variant, limiting statistical power to detect associations with the most commonly damaged regions, or on one language task (e.g. confrontation naming^9,10^), limiting the generalizability of conclusions drawn from the sample.

The present study was designed to address these issues. The study's goal was to characterize the neural correlates of a broad range of language behavioural deficits in PPA by (i) including all three common variants of PPA; (ii) including a large sample of PPA patients; (iii) exploring whether common regions of atrophy across PPA variants contribute to task performance; and (iv) probing a wider set of language tasks than is typically tested.

## Materials and methods

### Participants

One hundred and eighteen patients and 61 healthy, age-matched controls were recruited within seven locations (Bonn, Göttingen, Erlangen, Homburg, Leipzig, Munich, Rostock, Tübingen, Ulm and Würzburg) of the German Consortium for Frontotemporal Lobar Degeneration^11^ (FTLD; http://www.ftld.de). For each centre, clinical evaluation and neuropsychological and language assessments were performed according to standard operating procedures. PPA diagnosis required progressive deterioration of mainly speech and language for **≥** 2 years, and PPA variant was diagnosed by the criteria of Gorno-Tempini *et al*.^2^ Patients not fulfilling a specific PPA subtype were classified as mixed-variant and excluded from the current study (*n* = 21), given that mixed variant PPA is far more heterogeneous than other variants. The study was conducted following the Declaration of Helsinki and approved by the local ethics committees of all centres. Participants provided written informed consent.

### Neuropsychological and language measures

**Figure 1:**
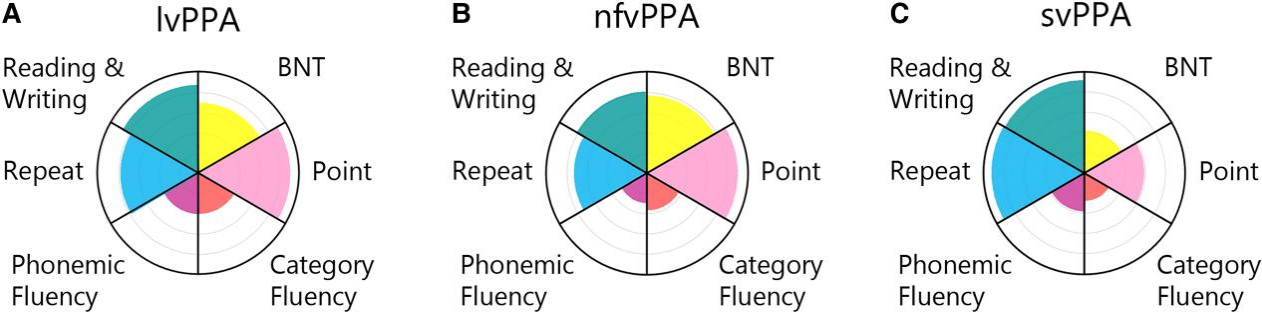
**Patient language behaviour.** PPA subgroup behaviour is shown as a proportion of control performance for logopenic variant PPA (**A**), nonfluent variant PPA (**B**) and semantic variant PPA (**C**).

The degree of general clinical impairment of the patients was assessed using the clinical dementia rating scale (sum of boxes, CDR-SB), FTLD-CDR-SB and mini-mental state exam (MMSE). Language measures included six tasks: the Boston naming test^12^ (BNT-15), a German adaptation of the Repeat and Point task,^13^ phonemic and category fluency tasks^14^ and the reading/writing subtest from the Aachen Aphasia Test^15^ (AAT; includes words, phrases and sentences). Table 1 shows patient and control averages on these measures, and Fig. 1 shows the performance of each PPA variant relative to controls. Tasks were administered and scored according to standard instructions. Tests of motor control and grammatical abilities were not administered due to testing time limitations and to limit the burden on patients, but clinical ratings of these skills broken down by subtype are included in Supplementary Tables 1–3. These ratings show that diagnosis of motor speech disorder and the presence of articulation and prosody difficulties were relatively higher in the nfvPPA group. Syntax difficulties were less discriminatory in our participants.

**Table 1:** Demographic and clinical characteristics of PPA variants and healthy controls

Variable	lvPPA	nfvPPA	svPPA	Controls	Group comparison
Number	22	51	45	61	
Gender (f/m)	13/9	22/29	22/23	32/29	
Age (years)	68.55 ± 6.28	67.59 ± 8.28	62.71 ± 6.72^a,b^	63.51 ± 11.64	H(3) = 13.31, *P* = 0.004
Disease duration	3.73 ± 3.27 (0)	2.31 ± 1.26 (2)^c^	4.02 ± 3.37 (4)		H(2) = 8.24, *P* = 0.016
CDR	2.98 ± 2.99 (1)***	2.92 ± 2.39 (7)***	4.34 ± 3.19 (4)***	0.03 ± 0.12 (12)	H(3) = 89.07, *P* < 0.001
FTLD-CDR	5.2 ± 3.58 (1)***	4.95 ± 3.14 (7)***	6.8 ± 4.23 (4)***	0.05 ± 0.15 (9)	H(3) = 108.78, *P* < 0.001
MMSE	22.09 ± 6.28 (0)***	23.46 ± 6.76 (1)***	20.86 ± 7.33 (3)***	29.12 ± 0.89 (4)	H(3) = 78.1, *P* < 0.001
Boston naming test	10.41 ± 3.32 (0)***	11.51 ± 3.67 (2)***	6.20 ± 3.31 (4)***^,a,b^	14.86 ± 0.44 (4)	H(3) = 112.54, *P* < 0.001
Point	8.81 ± 1.78 (1)**	8.70 ± 1.55 (7)***	5.63 ± 2.66 (4)***^,a,b^	9.76 ± 0.62 (19)	H(3) = 71.77, *P* < 0.001
Repeat	7.86 ± 2.50 (1)***^,c^	7.33 ± 3.05 (8)***^,c^	9.29 ± 1.45 (4)***	10 ± 0 (19)	H(3) = 50.13, *P* < 0.001
Phonemic fluency	6.86 ± 3.55 (1)***	5.02 ± 4.35 (5)***	6.56 ± 4.18 (6)***	17.11 ± 4.29 (5)	H(3) = 96.95, *P* < 0.001
Category fluency	11.05 ± 6.58 (0)***	9.65 ± 6.70 (2)***	7.41 ± 4.56 (4)***	26.80 ± 5.86 (5)	H(3) = 103.61, *P* < 0.001
Reading & writing (AAT)	77.55 ± 15.71 (0)***	71.78 ± 23.23 (15)***	80.97 ± 11.13 (9)***	88.31 ± 9.40 (19)	H(3) = 63.79, *P* < 0.001

Values shown are mean ± SD, with number of missing cases in parentheses.

a Score lower than lvPPA.

b Score lower than nfvPPA.

c Score lower than semantic PPA.

** *P* ≤ 0.01, compared to controls.

*** *P* < 0.001.

### Image acquisition

All structural images were acquired on Siemens Magnetom 3T or GE Signa HDxt scanners. T_1_-weighted images were acquired using a magnetization-prepared rapid gradient-echo sequence. Scanner parameters differed slightly across data collection sites (see Supplementary Table 4), so scanner was included as a covariate in all analyses.

### Statistical analysis

#### Clinical characteristics and behaviour

We used R version 4.0.0^16^ to compute statistics on behaviour. Healthy controls and PPA group differences were tested with two-tailed 1 × 4 Kruskal–Wallis tests, and follow-up tests were Bonferroni-corrected Mann–Whitney U tests.

#### Structural image processing and analysis

Images were processed with the CAT12 toolbox version 12.7 (http://www.neuro.uni-jena.de/cat/) in SPM12 (Wellcome Department of Imaging Neuroscience, London, UK; http://www.fil.ion.ucl.ac.uk/spm/software/spm12/) running in a MATLAB 9.7.0 (R2019b) environment (Mathworks, Inc., Sherbon, MA, USA). After a strong inhomogeneity correction, MRI images were segmented into grey matter, white matter and cerebrospinal fluid based on an adaptive maximum posterior technique^17,18^ that takes into account intensity inhomogeneity and other local variations of intensity. Volumetric images were then normalized to the standard Montreal Neurological Institute template including affine and non-linear modulation to account for local compression and expansion during transformation. SPM processing accuracy was set to high. After voxel-based processing was complete, surface-based processing estimated cortical thickness using a projection-based thickness method.^19^ Surface images were normalized to the Freesurfer ‘FsAverage’ template using a spherical mapping with minimal distortions.^18^ Cortical thickness maps were smoothed with a 20 mm kernel. The CAT12 toolbox provides several ways of assessing the quality of segmented volumes, including a weighted image quality grade and mean inter-image correlations. Six images were excluded from analysis due either to a low image quality grade (79% or worse; *n* = 4) or for being an outlier on other quality measures (*n* = 2). The excluded scans were not predominantly from any one PPA subgroup (two lvPPA, three nfvPPA and one svPPA). The final cohort excluding these scans included 118 patients.

Pairwise comparisons between the three PPA groups and healthy controls were performed in SPM12 using two-sample *t*-tests. To investigate structural–behavioural relationships, we used multivariable linear regression to predict cortical thickness with language task scores across all PPA patients. Patient groups were combined because clinico-anatomic correlations were not expected to differ by PPA variant, and increased variability of dependent variables was expected to increase statistical power.

Age, gender and scanner type (individual binary variables) were entered as covariates in the general linear model for all analyses. Overall grey matter volume (GMV) was also controlled in regressions with AAT reading and writing, as this task alone showed a correlation with GMV after Bonferroni correction. Statistically significant clusters were thresholded at FWE *P* < 0.05. Results from analyses using a less stringent threshold of FDR *P* < 0.05 are also available in Supplementary Figs 1 and 2.

Parallel analyses were performed on the volumetric data derived from the above CAT12 processing using voxel-based morphometry (VBM). Methods and results from these analyses, which largely parallel those for cortical thickness, are available for comparison in the supplemental materials.

### Data availability

Group-level statistical maps are available at https://osf.io/h4r2f/. Participant-level data cannot be shared because of general data protection regulations, i.e. to ensure patients’ data privacy. However, authors can be contacted for further analyses upon request.

## Results

### Demographics & language processing task performance

Behavioural performance data and group differences are summarized in Table 1 and Fig. 1. Patients were impaired compared to controls in all language tasks (*P <* 0.05). Patients with svPPA were distinguished from those with lvPPA and nfvPPA by lower BNT-15 and Point task scores (*P* < 0.001) and higher Repeat scores (*P* < 0.01).

### How is language performance related to regional atrophy in PPA?

**Figure 2:**
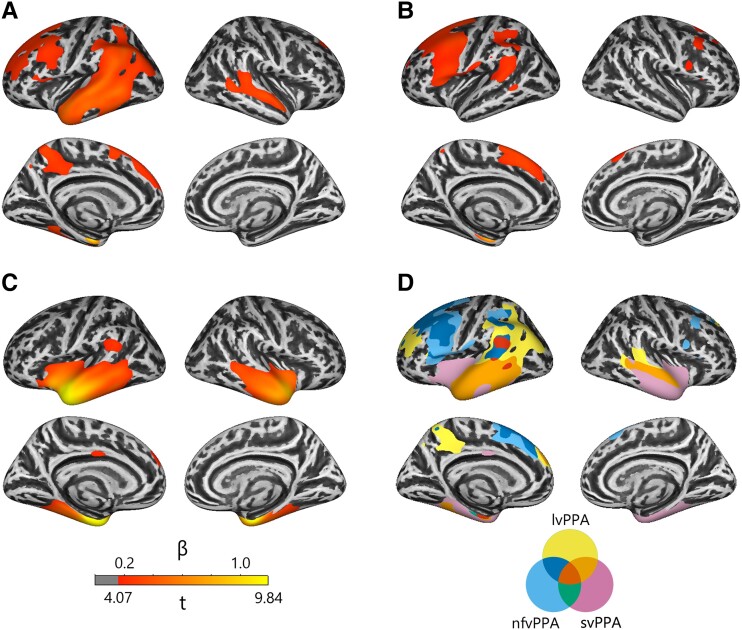
**PPA atrophy profiles.** PPA atrophy compared to controls is shown for logopenic variant PPA (**A**), nonfluent variant PPA (**B**) and semantic variant PPA (**C**). The overlap of atrophy across PPA variants is shown in **D**. All values shown are significant at cluster FWE *P* < 0.05.

**Figure 3:**
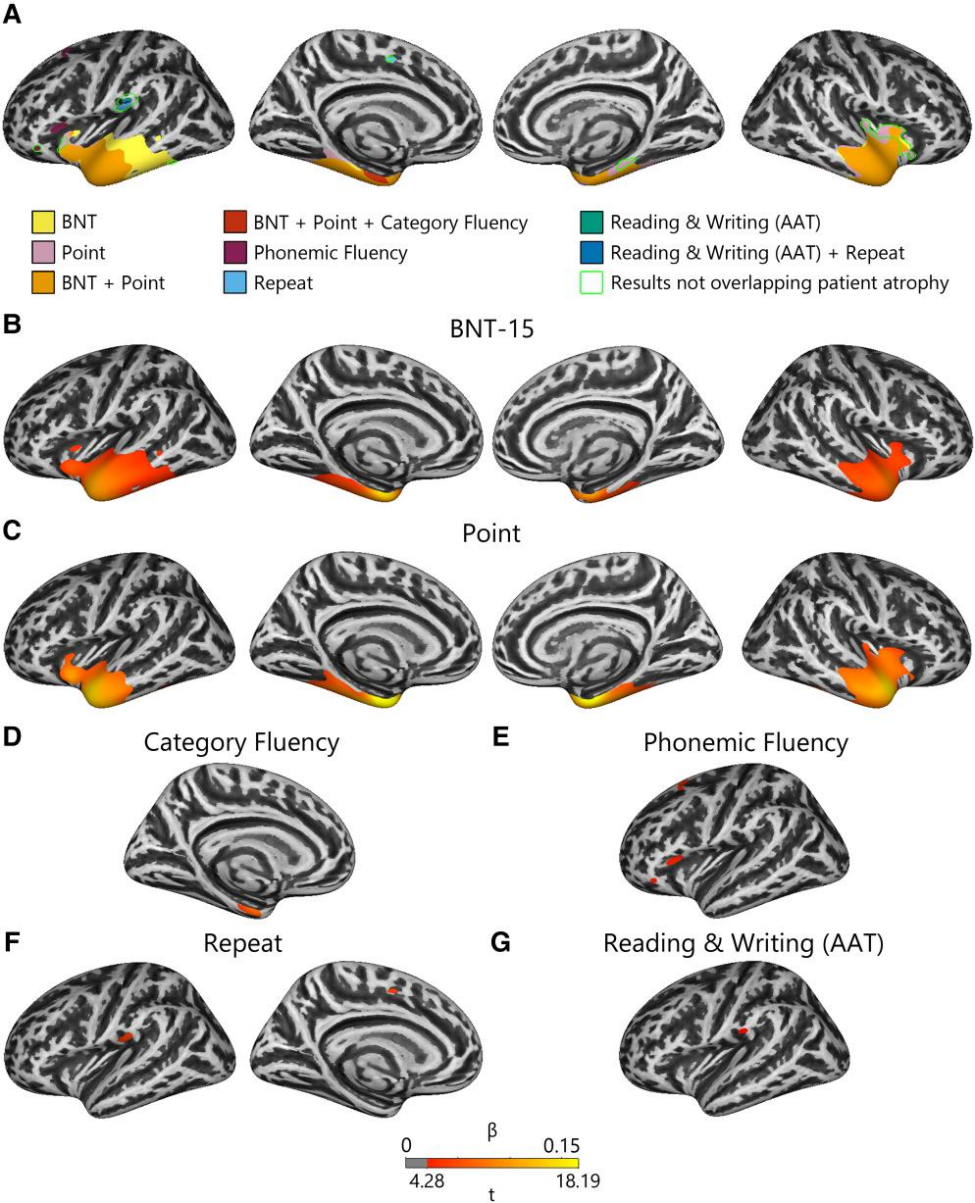
**Language behaviour regression results.** Regression of language behaviour with cortical thickness is shown for the overlap between all results (**A**) and for each task individually (**B**–**G**). All values shown are significant at cluster FWE *P* < 0.05.

PPA atrophy compared to controls is shown in Fig. 2, and significant associations between atrophy and behaviour are shown in Fig. 3 (see Supplementary Tables 5 and 6 for cluster maxima coordinates). All regions in cortical thickness analyses were significant at FWE *P* < 0.05.

Impaired performance on the BNT-15 and Point tasks was associated with atrophy to the bilateral temporal lobes extending medially. Performance on both tasks was associated with the temporal pole; inferior (ITG), middle (MTG) and superior temporal gyri (STG); entorhinal cortex; and anterior insula. Point performance was associated with a less posterior extent of left temporal cortex and a greater extent of right insular cortex.

Impaired Repeat performance was associated with atrophy to left medial superior frontal gyrus/posterior cingulate cortex and left postcentral gyrus.

Phonemic fluency performance was associated with left inferior frontal gyrus (IFG), as well as left lateral superior frontal gyrus (SFG).

Impaired performance in category fluency was associated with medial ATL centred on entorhinal cortex.

Impaired performance on reading/writing from the AAT was associated with left precentral gyrus.

### Which language-related regions are also atrophied in PPA?

**Figure 4:**
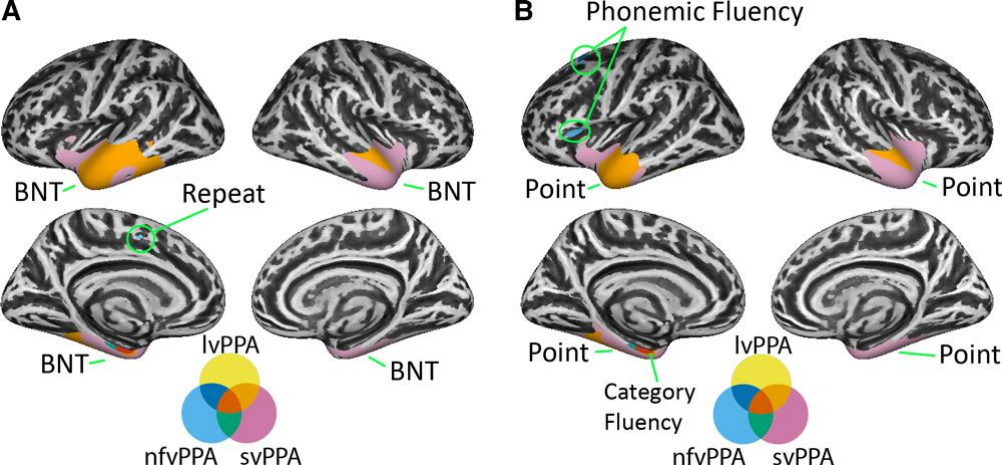
**Overlap of patient atrophy with language regression results. A** shows PPA atrophy overlapping language regression results for BNT and Repeat tasks. **B** shows PPA atrophy overlapping language regression results for Point, category fluency and phonemic fluency. No overlap was observed for reading and writing (AAT) with atrophied regions.

Next, we investigated whether regions associated with task performance were also atrophied in each PPA subtype. This was done by masking the anatomical–behavioural correlations with patient atrophy masks (Fig. 4 and Supplementary Fig. 3). The percentage of task-associated vertices overlapping with atrophy in each PPA variant is shown in Supplementary Table 7 and noted in parentheses below. The outline in Fig. 3 (and Supplementary Fig. 4) shows that while many task-related regions overlapped with patient atrophy, some did not.

All PPA variants showed common atrophy (red) in the medial temporal lobe that was associated with BNT-15 and Point performance (1% of task-associated vertices). Patients with svPPA and lvPPA showed a larger region of overlapping atrophy in areas associated with these tasks than did other PPA variant pairs (28–41%). Patients with svPPA showed the most atrophy in task-associated regions (92–98%), followed by lvPPA (28–41%) and nfvPPA (1–2%). Behavioural deficits in PPA variants mirror these results, as svPPA performed most poorly, followed by lvPPA and nfvPPA (Table 1).

Patients with nfvPPA and lvPPA showed overlapping atrophy in left SFG that was associated with phonemic fluency (24%). Otherwise, only nfvPPA atrophy overlapped with task-associated regions (85%).

All PPA variants showed common atrophy in left medial temporal lobe that was associated with category fluency (48%). Large clusters of overlapping atrophy were observed between lvPPA and svPPA (68%) and between nfvPPA and svPPA (63%) in task-associated regions. The remainder of task-associated cortex overlapped only with svPPA atrophy (100%).

Regions related to performance on the Repeat task overlapped only with nfvPPA (10%), consistent with nfvPPA having the numerically strongest behavioural impairment in in this task.

Reading and writing-associated regions showed no overlap with PPA atrophy.

## Discussion

This study sheds light on the relationship between cortical atrophy and language task performance in the three main clinical variants of PPA. It is the largest to date to explore such relationships across tasks tapping multiple facets of language processing. We provide evidence that: (i) the three main PPA variants share atrophy in several regions that correlate with language processing, suggesting common underlying cognitive deficits that lead to language impairments; (ii) severe semantic deficits in PPA depend on specific regions of the medial and ventral temporal lobes, especially in the left hemisphere; and (iii) apparently non-atrophied brain regions contribute to task deficits in PPA. We first discuss our findings of brain structure-language behaviour relationships contributing to the understanding of pathomechanisms in PPA.

### Brain regions associated with task performance in PPA

The set of brain regions in which grey matter measures were associated with task performance in PPA provided a statistically powerful validation of previous observations. Below, we highlight where our results are consistent with previous results and use reverse inference^20^ to suggest potential cognitive abilities underlying the associations.

Performance on picture naming and word-picture matching tasks showed largely overlapping, positive associations with grey matter measures in bilateral temporal regions, consistent with previous work.^5,6,21^ Although both tasks may be affected by many cognitive impairments, the overlapping anatomical associations of these tasks suggest additionally that a common underlying cognitive component to the tasks, likely verbal semantics, is driving the association. Indeed, both the bilateral ATLs and the medial temporal lobes have been previously associated with semantic processing.^22-25^

More notably, performance in picture naming correlated with atrophy to the left insula. Such a relationship is rarely reported in PPA,^21^ but previous studies have shown associations between the insula and phonological processing in neuroimaging^26^ and aphasia studies.^27^ Thus, we reason that the insular region found in the current study may be involved in phonological processing.

We also found that phonemic fluency performance was associated with parts of left superior and ventrolateral frontal lobe. Similar ventral clusters were previously associated with phonemic fluency in nfvPPA^28^ and in healthy adults.^29^ The observed region has been associated with phonological/articulatory planning processes or lexical retrieval^30^ and verbal working memory. The observed middle frontal region has been associated with executive processes related to verbal fluency.^30^ Thus, the observed association may indicate a role for phonological planning and executive processes deficits in phonemic fluency performance of PPA.

Repetition and reading and writing tasks were associated with the inferior part of the postcentral gyrus and repetition with the left medial superior frontal gyrus, neither of which has a clear relation to language processing nor has been observed previously.

### Common atrophy, common deficits

We observed regions associated with both atrophy and task performance in PPA, indicating their crucial relevance to language deficits. In many cases, PPA variants shared atrophy to regions correlated with task performance, providing evidence for a partial overlap in the neural and cognitive components contributing to task deficits across variants.

Consistent with previous longitudinal studies,^4,31^ and a recent study comparing cortical thickness and mean diffusivity in PPA,^32^ all PPA variants shared atrophy to the temporal lobes, at least in the long term, and atrophy to these regions correlated with picture naming, word-picture matching and sometimes category fluency performance. A larger region of temporal atrophy shared by lvPPA and svPPA was associated with the same tasks. Based on evidence from patients with semantic dementia, stroke and herpes simplex encephalitis, lateral and ventral temporal regions are likely a principal storage site for perceptual or amodal semantic representations.^24,33,34^ This evidence converges with neuroimaging meta-analyses^35^ to suggest that PPA patients share a mild semantic deficit that causes impairments in picture naming, word-picture matching and semantically driven word retrieval, which may explain previous observations that all PPA variants show semantic interference effects.^36^

Clusters of the superior frontal lobe associated with performance on the phonemic fluency task overlapped with both lvPPA and nfvPPA atrophy, suggesting that a shared executive deficit may contribute to their fluency deficits.

### Severe semantic deficits depend on ventral and medial temporal lobes

Despite the fact that svPPA patients share more atrophy in semantic task-related regions with lvPPA than nfvPPA (Fig. 2D), the latter variants show no difference in semantic impairments (Table 1). Thus, certain brain regions uniquely damaged in svPPA and associated with semantic tasks must be crucial to severe semantic deficits. Our findings are novel in that evidence from the overlapping atrophy of PPA variants in relation to behavioural–anatomical associations has never previously been brought to bear on this topic. Likely candidate regions are the ventral and medial aspects of the temporal lobes, especially the fusiform gyrus. Damage to these regions causes similarly severe semantic deficits in herpes simplex encephalitis,^37^ and various research postulates that anterior fusiform is a ‘semantic hub’, which contains amodal semantic representations.^23,33,38^

### Non-atrophied regions suggest future network interference

Although all PPA variants were impaired across all language tasks in the current study, not all PPA variants showed significant atrophy in task-associated regions. Furthermore, some brain regions associated with task performance—predominately inferior frontal, insular and perisylvian regions (Fig. 3)—were not significantly atrophied in any PPA group. These results provide novel evidence that PPA patients’ deficits are related to the relative integrity of cortex in which no atrophy is detected. While that absence of atrophy at a given statistical threshold does not necessarily imply the absence of neurological damage, our results show that the behaviour associated with PPA syndromes should not only be understood as related to regions that are obviously damaged. Many possibilities exist for task relationships with these regions, including below-threshold neurological damage, functional reorganization and network effects. Some research has suggested that neurodegeneration progresses within specific functional or anatomical cortical networks.^39,40^ Non-atrophied regions that correlated with task performance in our study are consistent with this theory, as white matter pathways connect them to regions atrophied in PPA, and such regions have previously shown increased functional connectivity to regions of focal atrophy.^39^ Future studies might further investigate the role of these regions with functional MRI and positron emission tomography to investigate changes in perfusion or metabolism and diaschisis phenomena. Moreover, studies with positron emission tomography and relevant tracers such as for tau or amyloid might also enable access to histopathological underpinnings.

### Limitations

This study has several limitations. Although the sample size of our study improves upon most previous studies, the individual PPA groups were still not as large as might be statistically desirable. Future studies with larger samples will help to validate our results. Our study was not able to consider clinico-anatomical correlations related to the two main clinical components of nfvPPA, speech articulation and grammar processing. Furthermore, our study did not consider white matter integrity in patients, which plays an essential role in the pathophysiology of nfvPPA. The current study also did not examine longitudinal data, which could be useful concerning the evolving relationship of brain atrophy and task-relevant cortex. Recent work has also indicated that cortical mean diffusivity may be a more sensitive measure of disease severity than cortical thickness and therefore may provide a more sensitive measure of relationships between atrophy and behaviour.^32^ Finally, participants with PPA in the current study may have had increased overlap in brain atrophy partially due to being at relatively more advanced stages of their disease. Previous studies have suggested that macrostructural overlap should be lower in subgroups of participants with mild PPA,^4^ although it is notable that participants with PPA from Illan-Gala *et al*.,^32^ who had lower average CDR-FTLD-SB scores than our participants with PPA, showed more widespread cortical atrophy.

## Conclusion

Our results provide useful evidence on how language performance relates to brain atrophy across PPA variants, how common neural and cognitive components underlie language deficits across PPA variants and how atrophy is likely to progress in PPA. These results provide a basis for understanding the cortical networks affected across PPA variants and, through this understanding, pathways for treatment. Task-associated regions constitute promising targets for rehabilitative neurostimulation, which recent studies have successfully implemented to improve language processing in PPA.^41^ Furthermore, stimulation of regions contralateral to task-associated regions may also provide a pathway for future therapy, given recent research on cognitive compensation in PPA.^42^

## Supplementary Material

fcad076_Supplementary_Data

## Appendix

Collaborators from the FTLD Consortium Germany are: Annerose Engel, Gerdi Pfüller, Daniéle Pino, Frank Regenbrecht, Angelika Thöne-Otto, Timo Oberstein, Bernhard Landwehrmeyer, Jolina Lombardi, Elisa Semler and Jan Kassubek.

## Abbreviations

AAT =: Aachen Aphasia Test
ATL =: anterior temporal lobe
BNT =: Boston naming test
CDR =: clinical dementia rating
IFG =: inferior frontal gyrus
ITG =: inferior temporal gyrus
MFG =: middle frontal gyrus
MMSE =: mini-mental state exam
MTG =: middle temporal gyrus
PPA =: primary progressive aphasia
SFG =: superior frontal gyrus
SMG =: supramarginal gyrus
STG =: superior temporal gyrus
TIV =: total intracranial volume
