# Supplementary material for: Structural correlates of language processing in primary progressive aphasia
Source: Brain Commun. 2023 Mar 16;5(2):fcad076. doi: 10.1093/braincomms/fcad076 (PMC10066572; doi:10.1093/braincomms/fcad076)
Supplement: fcad076_Supplementary_Data [file fcad076_supplementary_data.docx]

# **Supplemental material for: "Structural Correlates of Language Processing in Primary Progressive Aphasia"**

# **Collaborator Affiliations**

Collaborators from the FTLD Consortium Germany are: Annerose Engel^1^, Gerdi Pfüller^1^, Daniéle Pino^1^, Frank Regenbrecht^1^, Angelika Thöne-Otto^1^, Timo Oberstein^2^, Bernhard Landwehrmeyer^3^, Jolina Lombardi^3^, Elisa Semler^3^, Jan Kassubek^3^.

Collaborator affiliations:

1 Department of Neurology, University of Leipzig Medical Center, Leipzig 04103, Germany

2 Department of Psychiatry and Psychotherapy, University Hospital Erlangen, Erlangen 91054, Germany

3 Department of Neurology, University of Ulm, Ulm 89081, Germany

# **Supplemental Methods and Results**

For voxel-based morphometry (VBM) analyses, normalized segmented grey matter density maps were smoothed with a Gaussian kernel of 8 mm full width at half maximum. VBM regressions were performed in the same manner as cortical thickness analyses, except that VBM analyses also covaried total intracranial volume (TIV). Where no results passed the strict FWE *p*<.05 threshold, uncorrected *p*<.001 was used and is noted. masking task-associated regions with PPA brain atrophy, we always used maps with the same statistical threshold.

## **Correlation of Language Performance with Atrophy**

VBM results for BNT-15 and Point tasks was similar to results for cortical thickness. Both tasks were associated with the bilateral temporal lobes extending medially, with VBM showing additional associations for both tasks in the hippocampal and surrounding cortex, the left amygdala and left orbital frontal cortex, and for Point in the right amygdala. Impaired performance on the Repeat task (p<.001) was associated with atrophy to left frontal and parietal cortex, including left frontal pole, inferior frontal gyrus (IFG), insula, pre- and post-central, middle and superior frontal, supramarginal gyrus (SMG), angular gyrus, and right precentral gyrus. Impaired performance in phonemic fluency was associated with atrophy in left medial superior frontal gyrus. Impaired performance in category fluency was associated with atrophy to left posterior MTG. Finally, impaired performance on reading and writing from the AAT (p<.001), controlling for GMV, was associated with atrophy to predominately left frontal and parietal regions, including precentral gyrus, MFG, and superior parietal lobe.

## **Which Language-Related Regions Are Also Atrophied in PPA?**

Note that the percentage of task-associated regions vertices overlapping with atrophy in each PPA variant is noted in parentheses, as in the main text. All PPA variants showed common atrophy (red) in VBM in left lateral, ventral, and the medial temporal lobe that was associated with BNT-15 and Point performance (3-4%). Patients with svPPA and lvPPA showed a larger region of overlapping atrophy in task-associated areas than other variant pairs (9-14%). Patients with svPPA showed the most atrophy in task-associated regions (97-100%), followed by lvPPA (9-14%) and nvPPA (4%).

Regions associated with phonemic fluency in VBM did not overlap with PPA atrophy.

All PPA variants showed common atrophy in left posterolateral temporal lobe that was associated with category fluency (24%). Large clusters of overlapping atrophy were observed between lvPPA and svPPA (41%) and between nfvPPA and svPPA (27%) in task-associated regions. The remainder of task-associated cortex overlapped only with svPPA atrophy (100%).

The repeat task (*p*<.001) showed overlapping atrophy between lvPPA and nfvPPA in the left MFG, SMG, and insula (27%). Other task-related regions overlapped with svPPA (2%), nfvPPA (6%), and lvPPA atrophy (32%).

Reading and writing-associated regions (p<.001) only overlapped with nfvPPA atrophy (1%).

# **Supplemental Tables**

**Supplemental Table 1: Prevalence of motor speech disorders - number (prop.)**

| **subtype** | **no** | **yes** |
| --- | --- | --- |
| lvPPA | 11 (0.50) | 4 (0.18) |
| nfvPPA | 12 (0.24) | 15 (0.29) |
| svPPA | 20 (0.44) | 7 (0.16) |
| Numbers in parentheses comprise the proportion of all patients of a given PPA subtype included in the study. Ratings were not available for all participants, so proportions do not sum to 1. | | |

**Supplemental Table 2: Clinical rating of articulation and prosody issues - number (prop.)**

| **subtype** | **none** | **slight** | **obvious** | **difficult** | **very difficult** |
| --- | --- | --- | --- | --- | --- |
| lvPPA | 6 (0.27) | 1 (0.05) | 1 (0.05) | 1 (0.05) | 0 (0) |
| nfvPPA | 5 (0.10) | 9 (0.18) | 8 (0.16) | 2 (0.04) | 2 (0.04) |
| svPPA | 14 (0.31) | 3 (0.07) | 0 (0) | 0 (0) | 1 (0.02) |
| Numbers in parentheses comprise the proportion of all patients of a given PPA subtype included in the study. Ratings were not available for all participants, so proportions do not sum to 1. | | | | | |

**Supplemental Table 3: Clinical rating of syntax issues - number (prop.)**

| **subtype** | **none** | **slight** | **obvious** | **difficult** | **very difficult** |
| --- | --- | --- | --- | --- | --- |
| lvPPA | 5 (0.23) | 1 (0.05) | 0 (0) | 1 (0.05) | 2 (0.09) |
| nfvPPA | 10 (0.20) | 5 (0.10) | 6 (0.12) | 2 (0.04) | 2 (0.04) |
| svPPA | 7 (0.16) | 7 (0.16) | 2 (0.04) | 0 (0) | 2 (0.04) |
| Numbers in parentheses comprise the proportion of all patients of a given PPA subtype included in the study. Ratings were not available for all participants, so proportions do not sum to 1. | | | | | |

**Supplemental Table 4: Scanner parameters across data collection sites**

| **City** | **Scanner** | **Repetition time (ms)** | **Echo time (ms)** | **Flip angle** | **Slice thickness (mm)** | **Bandwidth (Hz/pixel)** | **Field of view** | **n Control** | **n lvPPA** | **n nfvPPA** | **n svPPA** |
| --- | --- | --- | --- | --- | --- | --- | --- | --- | --- | --- | --- |
| Bonn | Skyra | 2300 | 3.06 | 9 | 1 | 230 | 240*256 | 3 | 4 | 3 | 5 |
| Bonn | Skyra | 2500 | 4.82 | 7 | 1 | 140 | 256*256 | 0 | 0 | 1 | 0 |
| Erlangen | TrioTim | 1300 | 3.46 | 10 | 1 | 190 | 240*256 | 1 | 0 | 0 | 0 |
| Erlangen | TrioTim | 2300 | 2.98 | 9 | 1 | 238 | 240*256 | 3 | 0 | 2 | 0 |
| Erlangen | TrioTim | 2300 | 2.98 | 9 | 3 | 238 | 256*256 | 1 | 0 | 1 | 0 |
| Erlangen | TrioTim | 2300 | 2.98 | 9 | 5 | 238 | 256*256 | 0 | 1 | 0 | 0 |
| Goettingen | TrioTim | 2300 | 2.96 | 9 | 1 | 241 | 196*196 | 0 | 0 | 0 | 1 |
| Goettingen | TrioTim | 2300 | 2.96 | 9 | 1 | 241 | 201*201 | 0 | 0 | 1 | 0 |
| Goettingen | TrioTim | 2300 | 2.96 | 9 | 1 | 241 | 224*256 | 0 | 0 | 0 | 1 |
| Goettingen | TrioTim | 2300 | 2.96 | 9 | 1 | 241 | 232*256 | 0 | 0 | 0 | 1 |
| Goettingen | TrioTim | 2300 | 2.96 | 9 | 1 | 241 | 237*237 | 0 | 0 | 0 | 1 |
| Goettingen | TrioTim | 2300 | 2.96 | 9 | 1 | 241 | 240*256 | 2 | 1 | 3 | 0 |
| Goettingen | TrioTim | 2300 | 2.98 | 9 | 1 | 240 | 256*256 | 1 | 0 | 0 | 0 |
| Homburg | Skyra | 1900 | 3.29 | 9 | 0.9 | 210 | 240*240 | 0 | 0 | 0 | 1 |
| Homburg | Skyra | 2000 | 2.98 | 9 | 1 | 240 | 240*256 | 1 | 0 | 0 | 0 |
| Homburg | Skyra | 2300 | 2.98 | 9 | 1 | 238 | 256*256 | 3 | 0 | 1 | 0 |
| Homburg | Skyra | 2300 | 2.98 | 9 | 1 | 240 | 240*256 | 5 | 0 | 1 | 0 |
| Homburg | Skyra | 2300 | 2.98 | 9 | 4 | 240 | 256*256 | 0 | 1 | 0 | 0 |
| Leipzig | Biograph mmr | 1900 | 2.53 | 9 | 1 | 170 | 250*250 | 0 | 0 | 0 | 1 |
| Leipzig | Skyra | 2300 | 2.98 | 9 | 1 | 238 | 256*256 | 5 | 0 | 1 | 0 |
| Leipzig | TrioTim | 1930 | 4.3 | 18 | 1.5 | 210 | 256*256 | 0 | 1 | 0 | 0 |
| Leipzig | Verio | 1300 | 3.5 | 10 | 1 | 190 | 240*256 | 2 | 1 | 0 | 0 |
| Leipzig | Verio | 2300 | 2.98 | 9 | 1 | 238 | 240*256 | 2 | 1 | 1 | 4 |
| Leipzig | Verio | 2300 | 2.98 | 9 | 1 | 238 | 256*256 | 4 | 5 | 5 | 4 |
| Muenchen | Signa HDxt | 2300 | 2.98 | 9 | 1 | 238 | 256*256 | 0 | 1 | 0 | 3 |
| Muenchen | Signa HDxt | 6.62 | 3.15 | 15 | 3 | 139 | NA | 0 | 0 | 0 | 1 |
| Muenchen | Biograph mmr | 2300 | 2.98 | 9 | 1 | 238 | 240*256 | 4 | 0 | 5 | 8 |
| Muenchen | Verio | 2300 | 2.98 | 9 | 1 | 238 | 240*256 | 1 | 1 | 1 | 4 |
| Muenchen | Verio | 2300 | 2.98 | 9 | 1 | 238 | NA | 0 | 0 | 0 | 1 |
| Rostock | Skyra | 2500 | 4.82 | 7 | 1 | 140 | 256*256 | 0 | 0 | 1 | 0 |
| Rostock | Verio | 1900 | 2.52 | 9 | 1 | 170 | 250*250 | 2 | 0 | 0 | 0 |
| Rostock | Verio | 2500 | 4.82 | 7 | 1 | 140 | 256*256 | 11 | 1 | 9 | 3 |
| Tuebingen | Skyra | 2300 | 2.9 | 10 | 1 | 240 | 240*256 | 0 | 0 | 1 | 0 |
| Ulm | Allegra | 2200 | 4.38 | 8 | 1 | 130 | 256*256 | 5 | 1 | 8 | 4 |
| Ulm | Prisma | 2300 | 2.03 | 9 | 1 | 240 | 240*256 | 0 | 1 | 1 | 0 |
| Ulm | Prisma | 2300 | 2.05 | 9 | 1 | 240 | 240*256 | 3 | 0 | 0 | 0 |
| Wuerzburg | TrioTim | 2300 | 2.98 | 9 | 1 | 238 | 240*256 | 0 | 2 | 4 | 1 |

lvPPA = logopenic variant PPA; nfvPPA = nonfluent variant PPA; svPPA = semantic variant PPA.

**Supplemental Table 5: Cortical thickness analysis maxima**

| **Analysis** | **Cluster extent (vertices)** | **t** | **p** | **Location** | **x** | **y** | **z** |
| --- | --- | --- | --- | --- | --- | --- | --- |
| Control > lvPPA | 31290 | 9.83 | FWE < .05 | L MTG | -64 | -49 | 3 |
|  |  | 8.65 |  | L STG | -62 | -52 | 17 |
|  |  | 8.59 |  | L STG | -56 | -23 | -4 |
|  | 5401 | 7.02 |  | R STG | 51 | -11 | -12 |
|  |  | 6.24 |  | R STG | 53 | 5 | -18 |
|  |  | 6.22 |  | R MTG | 66 | -44 | 3 |
|  | 11892 | 6.75 |  | L MFG | -30 | 41 | 29 |
|  |  | 6.51 |  | L MFG | -23 | 56 | 23 |
|  |  | 6.49 |  | L SFG | -19 | 35 | 37 |
|  | 2870 | 5.9 |  | L Precuneus | -4 | -42 | 49 |
|  |  | 5.6 |  | L Precuneus | -4 | -38 | 40 |
|  |  | 5.58 |  | L Precuneus | -4 | -54 | 57 |
|  | 427 | 5.17 |  | L Entorhinal | -20 | -3 | -27 |
|  | 275 | 4.9 |  | R SFG | 20 | 41 | 41 |
|  | 536 | 4.86 |  | L IFG (p. Opercularis) | -36 | 16 | 7 |
|  |  | 4.6 |  | L IFG (p. Opercularis) | -54 | 23 | 5 |
|  | 20 | 4.56 |  | L Fusiform Gyrus | 47 | -19 | -31 |
|  | 46 | 4.52 |  | L Precuneus | -6 | -73 | 36 |
| Control > nfvPPA | 17089 | 7.55 | FWE < .05 | L MFG | -23 | 0 | 66 |
|  |  | 6.71 |  | L MFG | -34 | 22 | 11 |
|  |  | 6.34 |  | L SFG | -38 | 4 | 55 |
|  | 3116 | 5.67 |  | L Supramarginal Gyrus | -54 | -43 | 22 |
|  |  | 5.29 |  | L Supramarginal Gyrus | -60 | -47 | 10 |
|  |  | 5.22 |  | L STG | -65 | -41 | 11 |
|  | 447 | 5.3 |  | L Entorhinal | -19 | -7 | -20 |
|  | 3712 | 5.22 |  | L Superior Parietal | -48 | -36 | 49 |
|  |  | 5.06 |  | L Postcentral Gyrus | -37 | -38 | 44 |
|  |  | 5.03 |  | L Superior Parietal | -50 | -54 | 49 |
|  | 753 | 5.2 |  | R MFG | 45 | 15 | 41 |
|  |  | 4.93 |  | R SFG | 31 | 24 | 52 |
|  |  | 4.92 |  | R SFG | 37 | 18 | 46 |
|  | 361 | 5.12 |  | R IFG (p. Opercularis) | 48 | 8 | 17 |
|  | 229 | 5.05 |  | L MTG | -65 | -46 | 1 |
|  | 1209 | 4.74 |  | R SFG | 22 | 21 | 62 |
|  |  | 4.67 |  | R SFG | 4 | 24 | 55 |
|  |  | 4.41 |  | R SFG | 11 | 7 | 70 |
|  | 128 | 4.6 |  | R SFG | 23 | 36 | 41 |
|  | 132 | 4.58 |  | L MFG | -26 | 58 | 18 |
|  | 84 | 4.55 |  | L Precuneus | -3 | -55 | 56 |
|  | 41 | 4.36 |  | R MFG | 41 | 24 | 8 |
|  | 25 | 4.33 |  | L Supramarginal Gyrus | -62 | -31 | 19 |
| Control > svPPA | 25516 | 18.19 | FWE < .05 | L Temporal Pole | -41 | 13 | -38 |
|  |  | 16.05 |  | L ITG | -47 | -7 | -45 |
|  |  | 15.34 |  | L ITG | -38 | -15 | -36 |
|  | 17086 | 10.6 |  | R STG | 41 | 19 | -22 |
|  |  | 9.01 |  | R ITG | 33 | -5 | -44 |
|  |  | 8.94 |  | R ITG | 36 | 4 | -42 |
|  | 1265 | 5.54 |  | L Supramarginal Gyrus | -62 | -50 | 16 |
|  |  | 5.36 |  | L STG | -56 | -42 | 21 |
|  | 206 | 4.82 |  | L PCC | -5 | -6 | 32 |
|  | 314 | 4.69 |  | L SFG | -4 | 54 | 28 |
| Boston Naming | 17619 | 9.84 | FWE < .05 | L ITG | -46 | -5 | -37 |
|  |  | 9.77 |  | L MTG | -54 | 7 | -10 |
|  |  | 9.59 |  | L MTG | -48 | 10 | -17 |
|  | 11172 | 6.64 |  | R MTG | 45 | 6 | -22 |
|  |  | 6.17 |  | R ITG | 38 | -24 | -27 |
|  |  | 5.9 |  | R ITG | 49 | -18 | -36 |
|  | 106 | 4.45 |  | L insula | -32 | 11 | 7 |
|  | 62 | 4.29 |  | L STS | -57 | -44 | -2 |
| Boston Naming (GMV contr.) | 18899 | 10.22 | FWE < .05 | L ITG | -50 | -4 | -41 |
|  |  | 10.1 |  | L STG | -53 | 14 | -15 |
|  |  | 9.1 |  | L Entorhinal | -23 | -13 | -34 |
|  | 11671 | 6.73 |  | R MTG | 39 | 17 | -24 |
|  |  | 6.66 |  | R MTG | 51 | 11 | -27 |
|  |  | 6.42 |  | R Fusiform Gyrus | 35 | -26 | -28 |
|  |  | 4.57 |  | L Insula | -35 | 9 | 2 |
| Point | 13785 | 7.96 | FWE < .05 | L Fusiform Gyrus | -38 | -17 | -25 |
|  |  | 7.52 |  | L Entorhinal | -31 | -3 | -37 |
|  |  | 7.5 |  | L Fusiform Gyrus | -31 | -39 | -25 |
|  | 14320 | 7.75 |  | R MTG | 51 | 13 | -20 |
|  |  | 7.26 |  | R ITG | 37 | -24 | -26 |
|  |  | 6.94 |  | R Entorhinal | 28 | -11 | -34 |
|  | 22 | 4.24 |  | L ITG | -49 | -46 | -19 |
| Point (GMV contr.) | 13815 | 7.71 | FWE < .05 | R Insula | 47 | 20 | -24 |
|  |  | 7.03 |  | R STG | 28 | -10 | -34 |
|  |  | 6.95 |  | R Fusiform Gyrus | 35 | -25 | -28 |
|  | 13293 | 7.68 |  | L Fusiform Gyrus | -39 | -17 | -32 |
|  |  | 7.37 |  | L Fusiform Gyrus | -26 | -41 | -20 |
|  |  | 7.26 |  | L ITG | -31 | -4 | -34 |
|  |  | 4.33 |  | R Fusiform Gyrus | 23 | -40 | -10 |
| Category Fluency | 561 | 4.84 | FWE < .05 | L Entorhinal | -23 | -13 | -26 |
| Category Fluency (GMV contr.) | 194 | 4.55 | FWE < .05 | L Entorhinal | -19 | -8 | -21 |
| Phonemic Fluency | 334 | 4.76 | FWE < .05 | L insula | -36 | 22 | 10 |
|  | 91 | 4.52 |  | L IFG (p. Orbitalis) | -44 | 33 | -16 |
|  | 188 | 4.35 |  | L SFG | -21 | 17 | 58 |
|  |  | 4.35 |  | L MFG | -25 | 14 | 50 |
| Phonemic Fluency (GMV contr.) | 463 | 4.64 |  | L MFG | -22 | 18 | 49 |
|  |  | 4.52 |  | L MFG | -22 | 18 | 60 |
|  | 92 | 4.53 |  | L STG | -41 | 28 | -15 |
|  | 182 | 4.51 |  | L Insula | -32 | 21 | 12 |
|  | 42 | 4.32 |  | L MFG | -25 | 42 | 35 |
| Repeat | 362 | 4.7 | FWE < .05 | L Postcentral Gyrus | -42 | -25 | 20 |
|  | 88 | 4.46 |  | L PCC | -9 | -1 | 46 |
| Repeat (GMV contr.) | 357 | 4.68 | FWE < .05 | L Postcentral Gyrus | -42 | -25 | 20 |
|  | 73 | 4.43 |  | L PCC | -9 | -1 | 46 |
| Reading & Writing | 516 | 5.04 | FWE < .05 | L Postcentral Gyrus | -55 | -26 | 18 |
|  | 444 | 4.72 |  | L STG | -52 | 6 | -1 |
|  | 141 | 4.56 |  | R IFG (p. Opercularis) | 61 | 3 | 15 |
| Reading & Writing (GMV contr.) | 147 | 4.21 | FWE < .05 | L Postcentral Gyrus | -49 | -25 | 16 |

Given coordinates are in fsaverage space. L = left; R = right; ITG = inferior temporal gyrus; MTG = middle temporal gyrus; STG = superior temporal gyrus; IFG = inferior frontal gyrus; MFG = middle frontal gyrus; SFG = superior frontal gyrus; PCC = posterior cingulate cortex; lvPPA = logopenic variant PPA; nfvPPA = nonfluent variant PPA; svPPA = semantic variant PPA.

**Supplemental Table 6: VBM analysis maxima**

| **Analysis** | **Cluster extent (vox)** | **t** | **p** | **Location** | **BA** | **x** | **y** | **z** |
| --- | --- | --- | --- | --- | --- | --- | --- | --- |
| Control > lvPPA | 14452 | 9.96 | FWE < .05 | L MTG | 21 | -63 | -21 | -6 |
|  |  | 9.94 |  | L MTG | 39 | -56 | -56 | 16 |
|  |  | 9.84 |  | L MTG | 22 | -52 | -26 | 0 |
|  | 2843 | 8.11 |  | R MTG | 22 | 52 | -8 | -15 |
|  |  | 7.99 |  | R MTG | 21 | 50 | -12 | -21 |
|  |  | 6.73 |  | R MTG | 21 | 51 | -22 | -14 |
|  | 27 | 6.44 |  | L Inferior Occipital Gyrus | 19 | -39 | -69 | -2 |
| Control > nfvPPA | 5241 | 8.13 | FWE < .05 | L Hippocampus |  | -34 | -28 | -15 |
|  |  | 7.79 |  | L Hippocampus |  | -32 | -15 | -10 |
|  |  | 7.48 |  | L MTG | 21 | -60 | -40 | 4 |
|  | 4841 | 7.68 |  | L IFG (p. Triangularis) | 13 | -32 | 24 | 6 |
|  |  | 7.66 |  | L Putamen/Insula |  | -24 | 16 | -4 |
|  |  | 7.54 |  | L Putamen/Insula |  | -28 | 14 | 6 |
|  | 3270 | 9.46 |  | L SFG | 6 | -22 | 10 | 54 |
|  |  | 8.87 |  | L MFG | 8 | -26 | 21 | 52 |
|  |  | 8.34 |  | L SFG | 8 | -16 | 21 | 50 |
|  | 491 | 6.93 |  | L MTG | 19 | -45 | -66 | 4 |
|  |  | 6.92 |  | L Inferior Occipital Gyrus | 19 | -38 | -68 | -2 |
|  |  | 6.67 |  | L Inferior Occipital Gyrus | 19 | -46 | -70 | 2 |
|  | 469 | 6.6 |  | R Insula | 13 | 39 | 24 | 2 |
|  |  | 6.51 |  | R IFG (p. Triangularis) | 46 | 42 | 32 | 8 |
|  |  | 5.85 |  | R IFG (operculum) |  | 27 | 21 | 6 |
|  | 291 | 6.62 |  | R Medial Temporal Pole | 38 | 50 | 2 | -27 |
|  |  | 6.55 |  | R Medial Temporal Pole | 38 | 51 | 4 | -24 |
|  | 259 | 6.3 |  | L IFG (p. Opercularis) |  | -38 | 0 | 30 |
|  |  | 6.09 |  | L Precentral Gyrus | 6 | -36 | 2 | 38 |
|  | 221 | 6.41 |  | L Medial Temporal Pole | 20 | -34 | -2 | -39 |
|  | 126 | 5.86 |  | L Temporal Pole | 47 | -39 | 18 | -16 |
|  | 120 | 6.43 |  | L Amygdala |  | -14 | -15 | -14 |
|  | 45 | 5.84 |  | L ITG (Temporal Pole) | 38 | -39 | 8 | -34 |
| Control > svPPA | 100190 | 21.5 | FWE < .05 | L Fusiform | 20 | -34 | -4 | -40 |
|  |  | 19.5 |  | L Medial Temporal Pole | 38 | -30 | 6 | -34 |
|  |  | 18.2 |  | L Medial Temporal Pole | 38 | -40 | 12 | -26 |
|  | 43 | 5.68 |  | R ITG | 19 | 44 | -62 | -6 |
|  |  | 5.46 |  | R Fusiform Gyrus | 37 | 42 | -57 | -12 |
| BNT | 32246 | 11.2 | FWE < .05 | L ITG | 20 | -34 | -8 | -36 |
|  |  | 10.2 |  | L PHG | 36 | -33 | -27 | -28 |
|  |  | 9.96 |  | L Temporal Pole | 38 | -39 | 8 | -33 |
|  | 11518 | 7.55 |  | R Temporal Pole | 38 | 28 | 18 | -32 |
|  |  | 7.44 |  | R PHG | 36 | 39 | -20 | -33 |
|  |  | 7.18 |  | R Temporal Pole | 38 | 38 | 10 | -33 |
| BNT (GMV contr.) | 29093 | 10.8 | FWE < .05 | L ITG | 20 | -34 | -8 | -36 |
|  |  | 9.8 |  | L PHG | 36 | -33 | -27 | -28 |
|  |  | 9.78 |  | L Temporal Pole | 38 | -39 | 8 | -33 |
|  | 6033 | 7.11 |  | R Temporal Pole | 38 | 28 | 18 | -32 |
|  |  | 7.08 |  | R PHG | 36 | 39 | -20 | -32 |
|  |  | 6.72 |  | R Temporal Pole | 38 | 38 | 10 | -33 |
| Point | 23157 | 9.26 | FWE < .05 | L PHG | 36 | -34 | -27 | -30 |
|  |  | 8.24 |  | L PHG | 36 | -28 | -10 | -30 |
|  |  | 8.04 |  | L Temporal Pole | 38 | -24 | -20 | -42 |
|  | 23516 | 8.25 |  | R Temporal Pole | 38 | 28 | 10 | -34 |
|  |  | 8.18 |  | R PHG | 36 | 38 | -16 | -30 |
|  |  | 8.15 |  | R Temporal Pole | 38 | 39 | 9 | -32 |
| Point (GMV contr.) | 15413 | 8.11 | FWE < .05 | L PHG | 36 | -34 | -27 | -30 |
|  |  | 7.59 |  | L PHG | 36 | -28 | -10 | -30 |
|  |  | 7.35 |  | L Temporal Pole | 38 | -24 | -20 | -42 |
|  | 8593 | 7.02 |  | R Temporal Pole | 38 | 28 | 10 | -34 |
|  |  | 6.97 |  | L Temporal Pole | 38 | 39 | 9 | -32 |
|  |  | 6.82 |  | R PHG | 36 | 38 | -16 | -30 |
|  |  | 5.83 |  | R ITG | 20 | 60 | -12 | -46 |
| Category Fluency | 41 | 6.01 | FWE < .05 | L Fusiform Gyrus | 37 | -52 | -44 | -6 |
| Category Fluency (GMV contr.) | 2007 | 5.12 | < .001 | L Fusiform Gyrus | 37 | -52 | -44 | -6 |
|  |  | 4.27 |  | L ITG | 20 | -57 | -39 | -34 |
|  |  | 3.89 |  | L ITG | 20 | -50 | -32 | -14 |
|  | 583 | 4.28 |  | L Putamen |  | -33 | -18 | -9 |
|  | 139 | 3.67 |  | L Orbital Frontal | 45 | -22 | 6 | -21 |
|  |  | 3.41 |  | L Orbital Frontal | 45 | -24 | 14 | -18 |
|  | 218 | 3.64 |  | L MTG | 19 | -66 | -64 | 4 |
|  |  | 3.61 |  | L MTG | 19 | -57 | -62 | 3 |
|  |  | 3.6 |  | L Lateral Occipital Cortex | 19 | -50 | -64 | 3 |
|  | 918 | 3.56 |  | L Fusiform Gyrus | 37 | -39 | -3 | -39 |
|  |  | 3.55 |  | L MTG | 19 | -54 | -4 | -28 |
|  |  | 3.5 |  | L ITG | 20 | -46 | -10 | -27 |
| Phonemic Fluency | 154 | 5.92 | FWE < .05 | L SFG | 8 | -3 | 26 | 51 |
|  |  | 5.69 |  | L SFG | 8 | -3 | 34 | 56 |
|  | 69 | 5.76 |  | L SFG | 8 | -6 | 34 | 40 |
|  | 24 | 5.64 |  | L SFG | 6 | -2 | 15 | 60 |
| Phonemic Fluency (GMV contr.) | 4658 | 5.54 | < .001 | L SFG | 8 | -3 | 26 | 51 |
|  |  | 5.5 |  | L SFG | 8 | -6 | 34 | 40 |
|  |  | 5.32 |  | L SFG | 6 | -2 | 15 | 60 |
|  | 727 | 4.43 |  | L Orbital Frontal | 45 | -33 | 26 | -2 |
|  |  | 4.03 |  | L Insula |  | -34 | 21 | 6 |
|  | 162 | 3.92 |  | R MFG | 8 | 51 | 9 | 58 |
|  |  | 3.72 |  | R MFG | 8 | 38 | 3 | 54 |
|  | 32 | 3.68 |  | L Precuneus |  | -9 | -63 | 42 |
|  | 76 | 3.64 |  | L SFG | 6 | -27 | 21 | 52 |
|  | 71 | 3.64 |  | L Frontal Pole | 10 | -40 | 40 | -9 |
|  | 278 | 3.62 |  | L Orbital Frontal | 45 | -14 | 21 | -18 |
|  |  | 3.58 |  | L Orbital Frontal | 45 | -4 | 27 | -21 |
|  | 31 | 3.6 |  | L ACC | 24 | -10 | 40 | 9 |
|  | 108 | 3.47 |  | R Frontal Pole | 10 | 45 | 63 | 4 |
|  |  | 3.46 |  | R Frontal Pole | 10 | 42 | 52 | -2 |
|  | 30 | 3.45 |  | R Frontal Pole | 10 | 39 | 44 | 9 |
|  | 53 | 3.45 |  | L SPL | 7 | -28 | -50 | 52 |
|  | 30 | 3.42 |  | L Frontal Pole | 10 | -34 | 54 | -3 |
| Repeat | 3987 | 6.38 | < .001 | L Precentral Gyrus | 4 | -66 | 16 | 24 |
|  |  | 4.32 |  | L Precentral Gyrus | 4 | -64 | 0 | 44 |
|  |  | 4.31 |  | L IFG (p. Opercularis) | 44 | -54 | 4 | 16 |
|  | 1035 | 5.2 |  | L SMG | 40 | -69 | -51 | 40 |
|  |  | 4.93 |  | L Angular Gyrus | 39 | -60 | -50 | 39 |
|  |  | 4.63 |  | L SMG | 40 | -63 | -40 | 24 |
|  | 26 | 4.51 |  | L Orbital Frontal | 45 | -12 | 22 | -34 |
|  | 34 | 4.19 |  | L Precentral Gyrus | 4 | 69 | 6 | 8 |
|  |  | 3.29 |  | R SFG | 6 | 63 | 6 | 14 |
|  | 173 | 4.17 |  | L Frontal Pole | 10 | -52 | 45 | -16 |
|  | 73 | 3.67 |  | L Fusiform Gyrus | 19 | -38 | -75 | -10 |
|  | 84 | 3.66 |  | L SFG | 6 | -32 | -12 | 51 |
|  | 46 | 3.6 |  | L IFG (p. Triangularis) | 45 | -39 | 34 | 4 |
|  | 20 | 3.48 |  | L IFG (p. Triangularis) | 45 | -58 | 27 | -3 |
|  | 68 | 3.46 |  | L Frontal Pole | 10 | -50 | 48 | 14 |
|  | 70 | 3.46 |  | L Frontal Pole | 10 | -34 | 48 | -2 |
|  | 35 | 3.42 |  | L Postcentral Gyrus | 5 | -6 | -36 | 60 |
| Repeat (GMV contr.) | 2436 | 6.3 | < .001 | L Precentral Gyrus | 4 | -66 | 16 | 24 |
|  |  | 4.23 |  | L IFG (p. Opercularis) | 44 | -54 | 4 | 16 |
|  |  | 4.12 |  | L MFG | 8 | -33 | 6 | 38 |
|  | 761 | 5.08 |  | L SMG | 40 | -69 | -51 | 40 |
|  |  | 4.78 |  | L Angular Gyrus | 39 | -60 | -50 | 39 |
|  |  | 4.59 |  | L SMG | 40 | -62 | -40 | 24 |
|  | 105 | 4.02 |  | L Frontal Pole | 10 | -52 | 45 | -16 |
|  |  | 3.43 |  | L Frontal Pole | 10 | -54 | 48 | -9 |
|  | 27 | 3.52 |  | L Fusiform Gyrus | 19 | -38 | -75 | -10 |
|  | 76 | 3.47 |  | L Insula |  | -57 | -20 | 15 |
|  | 34 | 3.45 |  | L SFG | 6 | -32 | -12 | 51 |
| Reading & Writing | 1174 | 5.53 | < .001 | L MFG | 8 | -57 | 18 | 44 |
|  |  | 3.94 |  | L MFG | 8 | -58 | 9 | 46 |
|  |  | 3.83 |  | L MFG | 8 | -42 | 18 | 32 |
|  | 1549 | 4.91 |  | L Precentral Gyrus | 4 | -64 | -6 | 50 |
|  |  | 4.89 |  | L Precentral Gyrus | 4 | -68 | 14 | 12 |
|  |  | 4.49 |  | L Precentral Gyrus | 4 | -68 | 14 | 24 |
|  | 825 | 4.87 |  | L SMG | 40 | -74 | -38 | 26 |
|  |  | 4.23 |  | L SMG | 40 | -68 | -52 | 42 |
|  |  | 4.17 |  | L SMG | 40 | -60 | -40 | 26 |
|  | 161 | 4.42 |  | L Frontal Pole | 10 | -30 | 50 | -4 |
|  | 1404 | 4.35 |  | L SPL | 7 | -30 | -44 | 46 |
|  |  | 4.23 |  | L SMG | 40 | -38 | -50 | 54 |
|  |  | 3.99 |  | L Postcentral Gyrus | 1 | -45 | -34 | 52 |
|  | 293 | 4.34 |  | L ACC | 24 | -8 | -9 | 39 |
|  | 32 | 4.15 |  | L Lateral Occipital Cortex | 19 | -28 | -94 | 36 |
|  | 372 | 4.05 |  | L IFG (p. Orbitalis) | 47 | -33 | 27 | -9 |
|  | 273 | 4 |  | R SMG | 6 | 63 | 6 | 10 |
|  | 45 | 3.97 |  | L Precentral Gyrus | 4 | 70 | 2 | 18 |
|  | 100 | 3.86 |  | L PCC | 31 | -9 | -64 | 38 |
|  | 232 | 3.82 |  | L IFG (p. Opercularis) | 44 | -44 | 12 | 2 |
|  | 55 | 3.73 |  | L Paracingulate | 24 | -14 | 34 | 34 |
|  | 41 | 3.72 |  | R Occipital Pole | 18 | 26 | -90 | 0 |
|  | 325 | 3.64 |  | L Precentral Gyrus | 4 | -32 | -20 | 39 |
|  |  | 3.59 |  | L SFG | 6 | -38 | -10 | 51 |
|  |  | 3.56 |  | L Precentral Gyrus | 4 | -30 | -14 | 45 |
|  | 78 | 3.6 |  | R Angular Gyrus | 39 | 45 | -58 | 24 |
|  | 63 | 3.51 |  | R ACC | 32 | 0 | 14 | 39 |
|  | 47 | 3.5 |  | L Fusiform Gyrus | 19 | -39 | -72 | -12 |
|  | 52 | 3.43 |  | L IFG (p. Orbitalis) | 47 | -46 | 44 | -8 |
|  | 20 | 3.36 |  | R Frontal Pole | 46 | 38 | 40 | 10 |
|  | 47 | 3.5 |  | L Fusiform Gyrus | 19 | -39 | -72 | -12 |
|  | 52 | 3.43 |  | L IFG (p. Orbitalis) | 47 | -46 | 44 | -8 |
|  | 20 | 3.36 |  | R Frontal Pole | 46 | 38 | 40 | 10 |
| Reading & Writing (GMV contr.) | 73 | 4.96 | < .001 | L MFG | 8 | -57 | 18 | 44 |
|  |  | 3.4 |  | L MFG | 8 | -58 | 9 | 46 |
|  | 57 | 4.61 |  | L Precentral Gyrus | 4 | -64 | -6 | 50 |
|  |  | 3.71 |  | L MFG | 8 | -58 | -3 | 54 |
|  | 308 | 3.63 |  | L SPL | 7 | -30 | -42 | 45 |
|  |  | 3.53 |  | L SPL | 7 | -39 | -48 | 54 |
|  |  | 3.43 |  | L SPL | 7 | -26 | -48 | 51 |
|  | 41 | 3.56 |  | R Precentral | 4 | 68 | 9 | 9 |

Given coordinates are in MNI space. L = left; R = right; ITG = inferior temporal gyrus; MTG = middle temporal gyrus; STG = superior temporal gyrus; IFG = inferior frontal gyrus; MFG = middle frontal gyrus; SFG = superior frontal gyrus; SPL = superior parietal lobe; SMG = supramarginal gyrus; ACC = anterior cingulate cortex; PCC = posterior cingulate cortex; BA = Brodmann's area; lvPPA = logopenic variant PPA; nfvPPA = nonfluent variant PPA; svPPA = semantic variant PPA.

**Supplemental Table 7: Significant atrophied voxels or vertices overlapping significant task-related regions**

| **Task** | **PPA group** | **Sig. Regression Voxels** | **PPA Sig. Voxels** | | **Sig. Regression Vertices** | **PPA Sig. Vertices** | |
| --- | --- | --- | --- | --- | --- | --- | --- |
|  |  |  | **#** | **%** |  | **#** | **%** |
| Boston Naming Test | lvPPA | 43764 | 6119 | 14% | 28959 | 11838 | 41% |
|  | nfvPPA |  | 1911 | 4% |  | 457 | 2% |
|  | svPPA |  | 43594 | 100% |  | 28377 | 98% |
|  | lvPPA-nfvPPA |  | 1597 | 4% |  | 284 | 1% |
|  | lvPPA-svPPA |  | 6117 | 14% |  | 11838 | 41% |
|  | nfvPPA-svPPA |  | 1911 | 4% |  | 457 | 2% |
|  | lvPPA-nfvPPA-svPPA |  | 1597 | 4% |  | 284 | 1% |
| Point | lvPPA | 46673 | 4020 | 9% | 28127 | 7977 | 28% |
|  | nfvPPA |  | 2070 | 4% |  | 413 | 1% |
|  | svPPA |  | 45176 | 97% |  | 25964 | 92% |
|  | lvPPA-nfvPPA |  | 1451 | 3% |  | 274 | 1% |
|  | lvPPA-svPPA |  | 4020 | 9% |  | 7977 | 28% |
|  | nfvPPA-svPPA |  | 2070 | 4% |  | 413 | 1% |
|  | lvPPA-nfvPPA-svPPA |  | 1451 | 3% |  | 274 | 1% |
| Category Fluency | lvPPA | 41 | 17 | 41% | 561 | 381 | 68% |
|  | nfvPPA |  | 11 | 27% |  | 352 | 63% |
|  | svPPA |  | 41 | 100% |  | 561 | 100% |
|  | lvPPA-nfvPPA |  | 10 | 24% |  | 267 | 48% |
|  | lvPPA-svPPA |  | 17 | 41% |  | 381 | 68% |
|  | nfvPPA-svPPA |  | 11 | 27% |  | 352 | 63% |
|  | lvPPA-nfvPPA-svPPA |  | 10 | 24% |  | 267 | 48% |
| Phonemic Fluency | lvPPA | 247 | 0 | 0% | 613 | 150 | 24% |
|  | nfvPPA |  | 0 | 0% |  | 522 | 85% |
|  | svPPA |  | 0 | 0% |  | 0 | 0% |
|  | lvPPA-nfvPPA |  | 0 | 0% |  | 150 | 24% |
|  | lvPPA-svPPA |  | 0 | 0% |  | 0 | 0% |
|  | nfvPPA-svPPA |  | 0 | 0% |  | 0 | 0% |
|  | lvPPA-nfvPPA-svPPA |  | 0 | 0% |  | 0 | 0% |
| Repeat | lvPPA | 5651 | 1821 | 32% | 450 | 0 | 0% |
|  | nfvPPA |  | 313 | 6% |  | 47 | 10% |
|  | svPPA |  | 102 | 2% |  | 0 | 0% |
|  | lvPPA-nfvPPA |  | 1552 | 27% |  | 0 | 0% |
|  | lvPPA-svPPA |  | 92 | 2% |  | 0 | 0% |
|  | nfvPPA-svPPA |  | 102 | 2% |  | 0 | 0% |
|  | lvPPA-nfvPPA-svPPA |  | 92 | 2% |  | 0 | 0% |
| Reading & Writing (AAT) – GMV controlled | lvPPA | 7141 | 11 | 0% | 1101 | 0 | 0% |
|  | nfvPPA |  | 52 | 1% |  | 0 | 0% |
|  | svPPA |  | 0 | 0% |  | 0 | 0% |
|  | lvPPA-nfvPPA |  | 7 | 0% |  | 0 | 0% |
|  | lvPPA-svPPA |  | 0 | 0% |  | 0 | 0% |
|  | nfvPPA-svPPA |  | 0 | 0% |  | 0 | 0% |
|  | lvPPA-nfvPPA-svPPA |  | 0 | 0% |  | 0 | 0% |

# **Supplemental Figures**

**Supplemental Figure 1: Patient atrophy at varying statistical thresholds.**


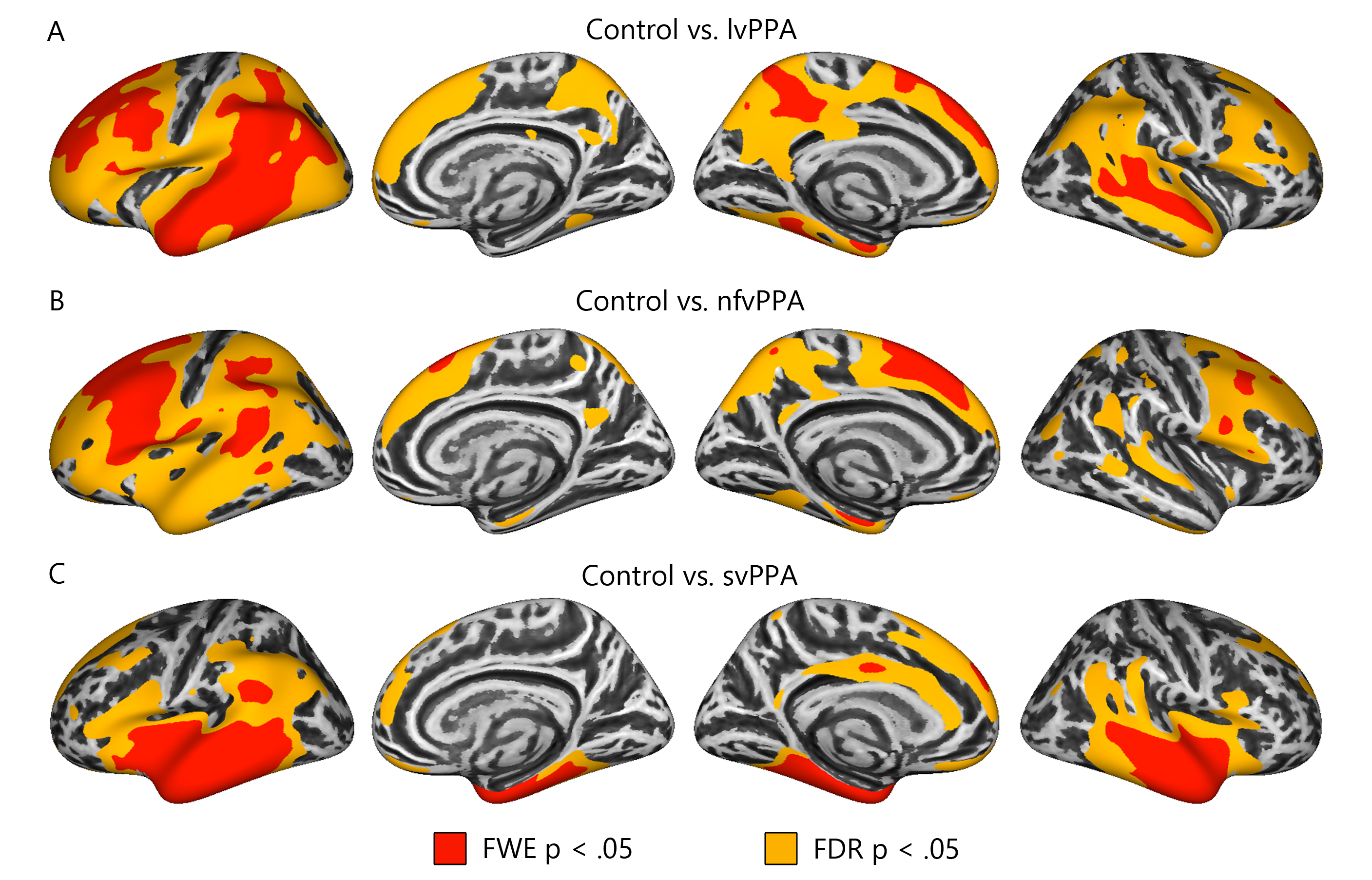


**Supplemental Figure 2: Language task regression results at FWE < .05 and
FDR < .05.**

**
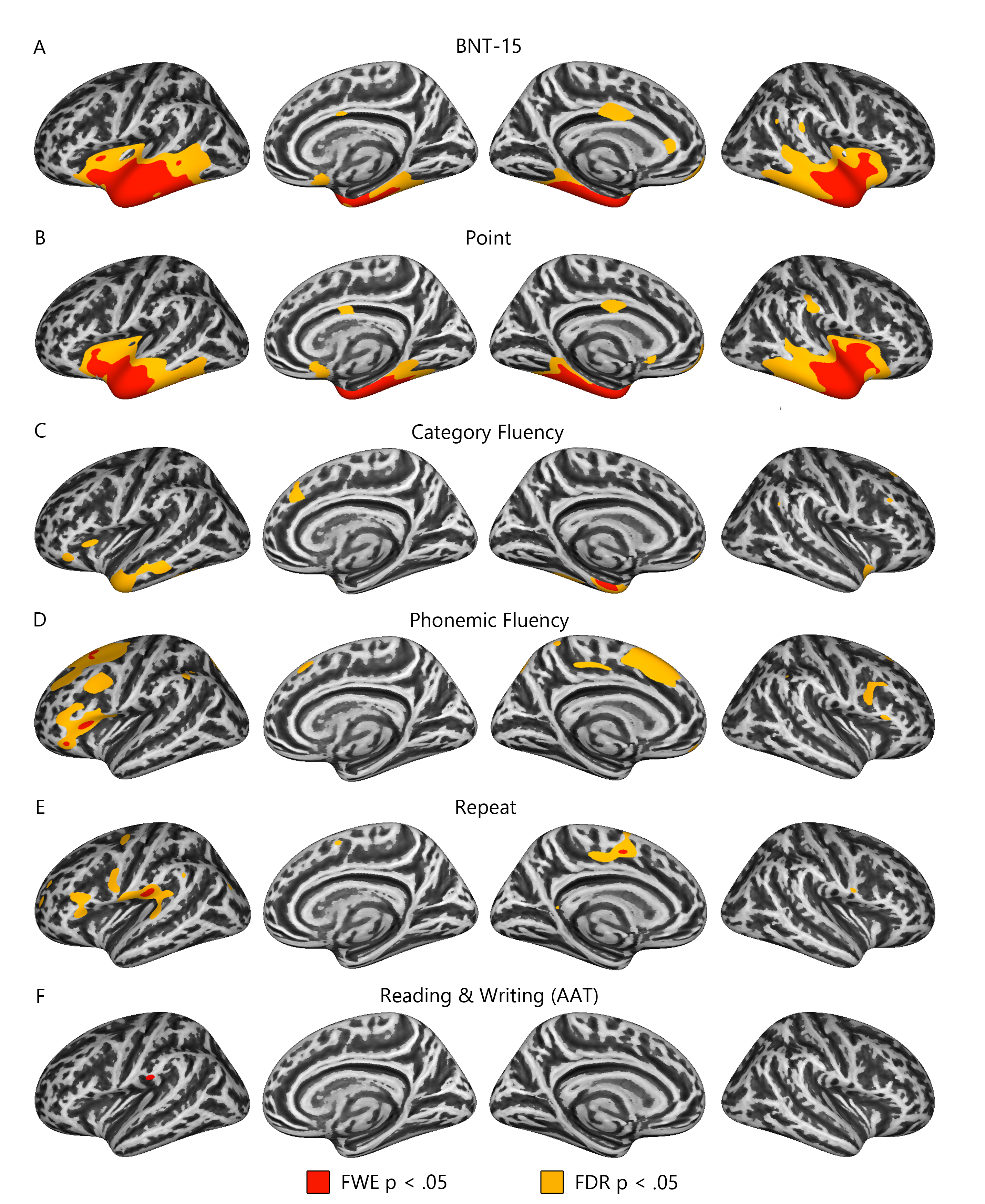
**

Language task regression results across PPA patients are shown at two different statistical thresholds for BNT-15 (A), Point (B), Category Fluency (C), Phonemic Fluency (D), Repeat (E), and Reading and writing (AAT) (F) tasks.

## **Primary Figures Including VBM and Cortical Thickness**

**Supplemental Figure 3: Overlap of patient atrophy with language behavior-related regions.**


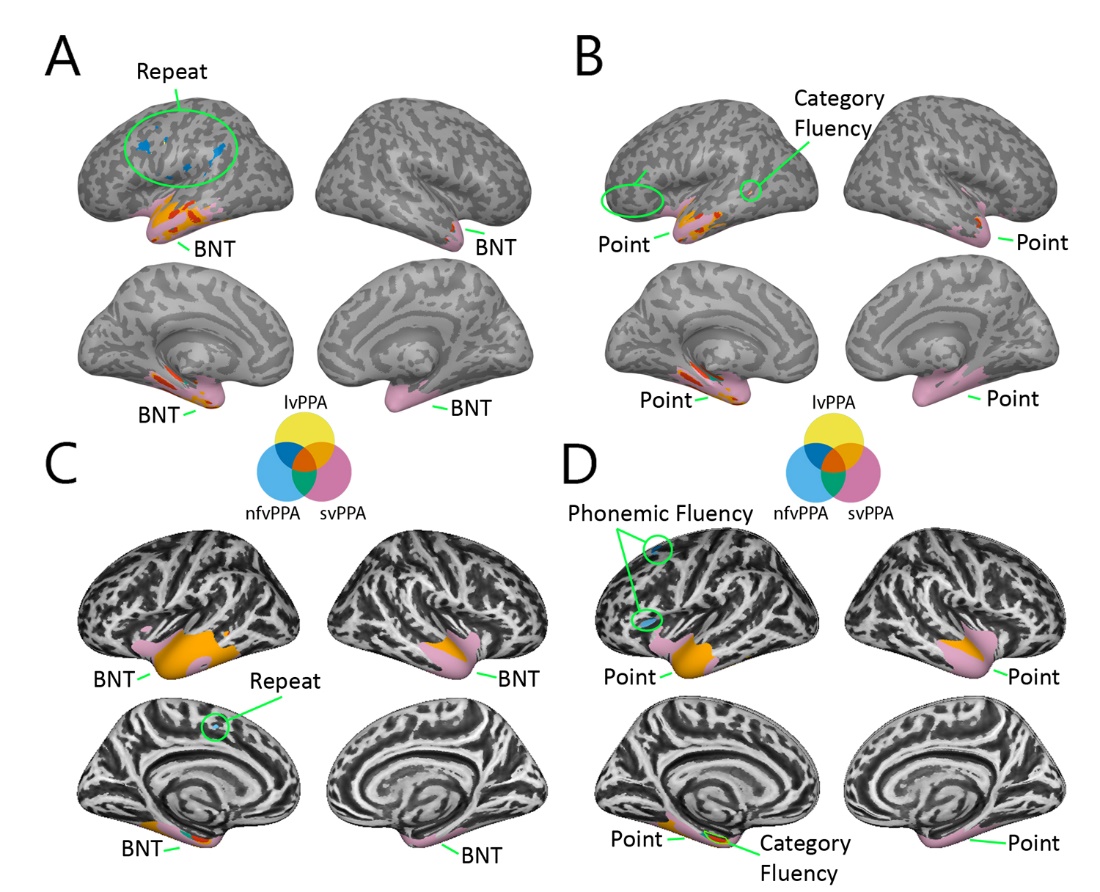


The left-hand column shows PPA atrophy overlapping the regression results for BNT and Repeat tasks for VBM (A) and cortical thickness (C). The right-hand column shows PPA atrophy overlapping regression results for Point, Category Fluency, and Phonemic fluency. Results for Reading & Writing (AAT) were not superficial enough to be visible in these plots.

**Supplemental Figure 4: Regions from regression analyses that did or did not overlap with PPA atrophy.**
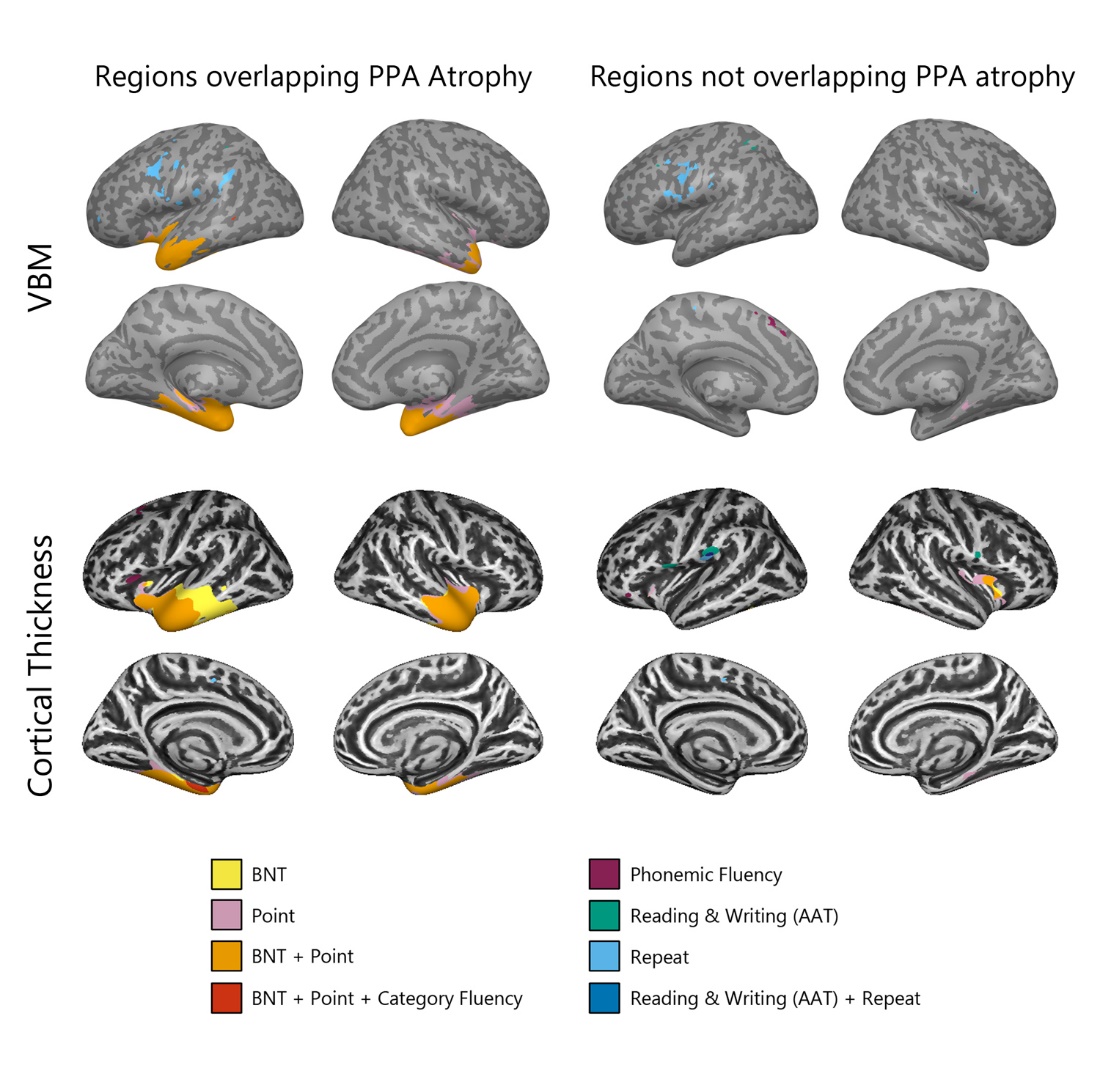


The left-hand column shows regions overlapping with PPA atrophy in VBM (top) and cortical thickness (bottom) analyses. The right-hand column shows regions not overlapping with PPA atrophy in VBM (top) and cortical thickness (bottom) analyses.
